# Prediction of High Nodal Burden in Patients With Sentinel Node–Positive Luminal *ERBB2*-Negative Breast Cancer

**DOI:** 10.1001/jamasurg.2024.3944

**Published:** 2024-09-25

**Authors:** Ida Skarping, Pär-Ola Bendahl, Robert Szulkin, Sara Alkner, Yvette Andersson, Leif Bergkvist, Peer Christiansen, Tove Filtenborg Tvedskov, Jan Frisell, Oreste D. Gentilini, Michalis Kontos, Thorsten Kühn, Dan Lundstedt, Birgitte Vrou Offersen, Roger Olofsson Bagge, Toralf Reimer, Malin Sund, Lisa Rydén, Jana de Boniface

**Affiliations:** 1 Division of Oncology, Department of Clinical Sciences, Lund University, Lund, Sweden; 2 Department of Clinical Physiology and Nuclear Medicine, Skane University Hospital, Lund, Sweden; 3 Cytel Inc, Sweden; 4 Department of Medical Epidemiology and Biostatistics, Karolinska Institutet, Sweden; 5 Department of Hematology, Oncology and Radiation Physics, Skane University Hospital, Lund, Sweden; 6 Department of Surgery, Västmanland Hospital, Västerås, Sweden; 7 Centre for Clinical Research Uppsala University, Västmanland Hospital Västerås, Sweden; 8 Centre for Clinical Research Uppsala University, Västmanland Hospital Västerås, Sweden; 9 Department of Plastic and Breast Surgery, Aarhus University Hosoital, Denmark; 10 Department of Breast Surgery, Gentofte Hospital, Denmark; 11 Faculty of Health and Medical Sciences, University of Copenhagen, Copenhagen, Denmark; 12 Department of Molecular Medicine and Surgery, Karolinska Institutet, Stockholm, Sweden; 13 Breast Center Karolinska, Karolinska Comprehensive Cancer Center, Karolinska University Hospital, Stockholm, Sweden; 14 Università Vita-Salute San Raffaele, Milano, Italy; 15 IRCCS Ospedale San Raffaele, Milano, Italy; 16 1st Department of Surgery, National and Kapodistrian University of Athens, Laiko Hospital, Athens, Greece; 17 Interdisciplinary Breast Center, University of Ulm, Ulm, Germany; 18 Breast Center Die Filderklinik, Filderstadt, Germany; 19 Department of Oncology, Institute of Clinical Sciences, Sahlgrenska Academy, University of Gothenburg, Gothenburg, Sweden; 20 Department of Oncology, Sahlgrenska University Hospital, Gothenburg, Sweden; 21 Department of Experimental Clinical Oncology, Aarhus University Hospital, Aarhus, Denmark; 22 Department of Oncology, Aarhus University Hospital, Aarhus, Denmark; 23 Aarhus University, Faculty of Health, Aarhus, Denmark; 24 Sahlgrenska Center for Cancer Research, Department of Surgery, Institute of Clinical Sciences, Sahlgrenska Academy, University of Gothenburg, Gothenburg, Sweden; 25 Department of Surgery, Sahlgrenska University Hospital, Gothenburg, Sweden; 26 Wallenberg Centre for Molecular and Translational Medicine, University of Gothenburg, Gothenburg, Sweden; 27 Department of Obstetrics and Gynecology, University of Rostock, Rostock, Germany; 28 Department of Surgery, University of Helsinki and Helsinki University Hospital, Helsinki, Finland; 29 Department of Diagnostics and Intervention/Surgery, Umeå University, Umeå, Sweden; 30 Department of Surgery and Gastroenterology, Skane University Hospital, Lund, Sweden; 31 Department of Surgery, Capio St Göran’s Hospital, Stockholm, Sweden; 32 Department of Molecular Medicine and Surgery, Karolinska Institutet, Stockholm, Sweden

## Abstract

**Importance:**

In patients with clinically node-negative (cN0) breast cancer and 1 or 2 sentinel lymph node (SLN) macrometastases, omitting completion axillary lymph node dissection (CALND) is standard. High nodal burden (≥4 axillary nodal metastases) is an indication for intensified treatment in luminal breast cancer; hence, abstaining from CALND may result in undertreatment.

**Objective:**

To develop a prediction model for high nodal burden in luminal *ERBB2*-negative breast cancer (all histologic types and lobular breast cancer separately) without CALND.

**Design, Setting, and Participants:**

The prospective Sentinel Node Biopsy in Breast Cancer: Omission of Axillary Clearance After Macrometastases (SENOMAC) trial randomized patients 1:1 to CALND or its omission from January 2015 to December 2021 among adult patients with cN0 T1-T3 breast cancer and 1 or 2 SLN macrometastases across 5 European countries. The cohort was randomly split into training (80%) and test (20%) sets, with equal proportions of high nodal burden. Prediction models were developed by multivariable logistic regression in the complete luminal *ERBB2*-negative cohort and a lobular breast cancer subgroup. Nomograms were constructed. The present diagnostic/prognostic study presents the results of a prespecified secondary analysis of the SENOMAC trial. Herein, only patients with luminal *ERBB2*-negative tumors assigned to CALND were selected. Data analysis for this article took place from June 2023 to April 2024.

**Exposure:**

Predictors of high nodal burden.

**Main Outcomes and Measures:**

High nodal burden was defined as ≥4 axillary nodal metastases. The luminal prediction model was evaluated regarding discrimination and calibration.

**Results:**

Of 1010 patients (median [range] age, 61 [34-90] years; 1006 [99.6%] female and 4 [0.4%] male), 138 (13.7%) had a high nodal burden and 212 (21.0%) had lobular breast cancer. The model in the training set (n = 804) included number of SLN macrometastases, presence of SLN micrometastases, SLN ratio, presence of SLN extracapsular extension, and tumor size (not included in lobular subgroup). Upon validation in the test set (n = 201), the area under the receiver operating characteristic curve (AUC) was 0.74 (95% CI, 0.62-0.85) and the calibration was satisfactory. At a sensitivity threshold of ≥80%, all but 5 low-risk patients were correctly classified corresponding to a negative predictive value of 94%. The prediction model for the lobular subgroup reached an AUC of 0.74 (95% CI, 0.66-0.83).

**Conclusions and Relevance:**

The predictive models and nomograms may facilitate systemic treatment decisions without exposing patients to the risk of arm morbidity due to CALND. External validation is needed.

**Trial Registration:**

ClinicalTrials.gov Identifier: NCT02240472

## Key Points

**Question:**

Can high nodal burden (≥4 axillary metastases/≥N2) be predicted without completion axillary lymph node dissection (CALND) in patients with luminal *ERBB2*-negative tumors and, separately, in those with invasive lobular carcinoma, and 1 or 2 sentinel lymph node (SLN) macrometastases?

**Findings:**

In this diagnostic/prognostic study including the CALND arm (n = 1010) of the randomized SENOMAC trial, 2 prediction models for the identification of patients with ≥N2 were developed, including number of SLN macrometastases, additional SLN micrometastases, SLN ratio, extracapsular extension, and tumor size (only luminal model).

**Meaning:**

The prediction models may be used to identify patients at risk of high nodal burden eligible for intensified systemic treatment strategies without performing CALND.

## Teaser

This diagnostic/prognostic study evaluates prediction models for high nodal burden in luminal *ERBB2*-negative breast cancer.

## Introduction

Sentinel lymph node (SLN) biopsy is the standard procedure for axillary staging in patients with clinically node-negative (cN0) breast cancer. While completion axillary lymph node dissection (CALND) was previously routine care in patients with positive SLNs, it is today safely omitted in patients with 1 or 2 SLN metastases.^1,2,3^ However, some systemic treatment recommendations in estrogen receptor (ER)–positive Erb-B2 receptor tyrosine kinase 2 (*ERBB2*)–negative breast cancer rely on the identification of high nodal burden (ie, ≥4 metastatic axillary lymph nodes [ALNs]), which can only be determined by CALND.^4^ The recent Abemaciclib Plus Endocrine Therapy for Hormone Receptor–Positive, HER2-Negative, Node-Positive, High-Risk Early Breast Cancer (MONARCHE) trial^5,6^ showed that patients with high-risk early ER-positive breast cancer, defined as 4 or more metastatic ALNs or 1 to 3 metastatic ALNs together with either tumor size of 5 cm or greater or histologic grade 3, had improved invasive disease-free survival after 2 years of adjuvant CDK 4/6 inhibitor treatment in addition to endocrine therapy compared to the control group. In the Microarray in Node-Negative and 1 to 3 Positive Lymph Node Disease May Avoid Chemotherapy (MINDACT) trial,^7^ axillary nodal burden was indicative of systemic treatment. In addition, screening for distant metastases and locoregional radiotherapy target extension are considered in breast cancer with high nodal burden.^8^ High nodal burden can thus alter adjuvant treatment decisions. Therefore, CALND omission may lead to undertreatment.

Negative outcomes of CALND, such as arm lymphedema (affecting up to 25% of patients), numbness, and restricted shoulder mobility, are well documented.^3^ Arm morbidity is considerably worse after CALND than after SLN biopsy only.^9,10^ Therefore, alternative strategies to correctly identify high nodal burden without exposing patients to the risk of long-term arm morbidity are warranted.

Known indicators of high nodal burden are larger tumor size and a higher number of suspicious nodes at axillary imaging.^11^ Risk factors for additional non-SLN metastases in patients with metastatic SLNs (ie, not necessarily a high nodal burden) also include a high ratio of metastatic to excised SLNs (SLN ratio), extracapsular extension, and lymphovascular invasion in the primary tumor.^12^

Invasive lobular carcinoma (ILC) is most often of luminal A subtype and accounts for 10% to 15% of all breast cancer cases.^13,14,15,16^ In comparison to invasive breast cancer of no special type (NST), ILC is associated with a higher nodal burden and a higher rate of non-SLN metastases.^17,18,19^ In ILC, axillary ultrasonography, fine-needle aspiration cytology, and core needle biopsy have a lower sensitivity for detection of nodal metastases than in NST.^20,21,22^ In addition, SLN biopsy has a higher false negative rate in ILC than in NST.^23^ Unfortunately, no published axillary de-escalation trials have presented subgroup analyses addressing ILC.^1,2,3^ Additionally, ILC is associated with late recurrences, which may not be fully captured by follow-up times ranging from 5.0 to 9.3 years.^1,2,3^

Prediction models aimed at detecting high nodal burden offer a tool to identify individuals who require intensified adjuvant treatments while avoiding CALND.^24,25,26,27,28^ However, existing models are based on retrospective data encompassing any histopathological and molecular subtypes. None of the models have focused on luminal breast cancer, where nodal burden influences adjuvant strategies and no distinction between NST and ILC has been made. The primary aim of the present study was to develop prediction models for high nodal burden in patients with 1 or 2 SLN macrometastases and luminal *ERBB2*-negative breast cancer and ILC, respectively, which could reduce the risk of undertreatment without exposing patients to CALND.

## Methods

### Patient Characteristics

The Sentinel Node Biopsy in Breast Cancer: Omission of Axillary Clearance After Macrometastases (SENOMAC) trial is a randomized (1:1), prospective, international, noninferiority trial. The study protocol was approved by the ethics committee at Karolinska Institutet, Stockholm, Sweden, and the trial was conducted according to good clinical practice. Enrollment was from January 2015 to December 2021. Randomized assignment was to CALND or its omission. Adjuvant treatment was according to national guidelines. Informed written consent was obtained from all individual participants prior to study inclusion.

The SENOMAC trial design and end points have been previously described, and initial results have been presented.^29,30^ Adult patients diagnosed with primary invasive cN0 T1-T3 breast cancer and 1 or 2 SLN macrometastases were eligible. Additional SLN micrometastases were allowed. Preoperative axillary ultrasonography was mandatory. While patients having an SLN biopsy prior to preoperative systemic therapy were eligible, 55 such patients were excluded from the present analysis since their CALND results, obtained after systemic therapy, may not reflect the initial nodal status. Overall, 1010 patients with luminal *ERBB2*-negative breast cancer randomized to CALND constituted the present study cohort, 212 of whom (21.0%) had ILC (eFigure in Supplement 1). Data analysis for this article took place from June 2023 to April 2024. The Transparent Reporting of a Multivariable Prediction Model for Individual Prognosis or Diagnosis (TRIPOD) reporting guideline was followed.

### Important Definitions

The TNM classification system was used. Thus, pathological nodal status (pN) was pN1 in case of 1 to 3 nodal metastases, pN2 for 4 to 9, and pN3 for 10 or more metastases.^31^ High nodal burden was defined as pN2 or pN3. Histopathological tumor type was categorized into 3 groups: NST, ILC, or other/mixed.^32^

### Statistical Analysis

Sample size calculations were performed for the primary and secondary outcomes of the SENOMAC trial, but not for the present analysis.^29^ Patient and tumor demographic characteristics were summarized using standard descriptive statistical measures. Differences between the subgroups were evaluated using χ^2^ tests for categorical variables, linear trend tests for characteristics measured on an ordinal scale, and *t* tests for continuous variables.

Two prediction models for pN2-3 vs pN1 were developed: one for the entire luminal *ERBB2*-negative cohort and another for the ILC subgroup. For the development of the prediction model for the luminal *ERBB2*-negative cohort, patients were randomly split into a training set (80%) and a test set (20%) for validation. The split was stratified on outcome (≥pN2) to get the same prevalence of high nodal burden in both datasets. To minimize the risk of overfitting, the prediction model was developed in the training set and evaluated in the test set. The ILC subgroup was not split due to its smaller size.

Routinely accessible variables after SLN biopsy were selected based on published literature and considered as candidates for the prediction models (eAppendix 1 in Supplement 1). Univariable logistic regression analysis was used to estimate associations between candidate predictors and high nodal burden in the luminal *ERBB2*-negative cohort and the ILC subgroup, respectively.

The model development steps are described in detail in eAppendix 1 in Supplement 1. Briefly, backward logistic regression analysis was combined with bootstrap to select a subset of predictor variables for the final model. Since the fraction of missing data was low, models were developed using data from patients with complete data.

The diagnostic performance of the prediction model for the luminal *ERBB2*-negative cohort was evaluated in terms of discrimination (area under the receiver operating characteristic curve [AUC]) and calibration (Hosmer-Lemeshow plots, calibration slope, and intercept). The model for the luminal *ERBB2*-negative cohort was validated in the test set. Since no test set was set aside for the ILC subgroup, this model was not internally validated and should be regarded as exploratory. The equations for the final prediction models are presented in eAppendix 2 in Supplement 1. Nomograms were created. Sensitivity, specificity, positive predictive value, negative predictive value, true negative, true positive, false negative, and false positive rates for the models were calculated for clinically relevant predetermined cutoffs.

The prognostic value of the model for the luminal *ERBB2*-negative cohort was evaluated with recurrence-free survival (RFS) as the end point, using Kaplan-Meier estimates together with 1-*df* log-rank test for trend over the risk groups, and Cox regression. RFS was defined according to the Standardized Definitions for Efficacy End Points (STEEP) criteria.^33^ The proportional hazards assumption in the Cox models was tested using Schoenfeld residuals.

The evidence against each null hypothesis was quantified by a *P* value and the word *significant* used for 2-sided *P* < .05. The reported *P* values have not been adjusted for multiple comparisons. R version 4.2.2 (R Foundation) was used for all statistical analyses.

## Results

### Descriptive Results

#### High vs Low Nodal Burden

Of 1010 patients with luminal *ERBB2*-negative breast cancer assigned to CALND in the SENOMAC trial (luminal cohort; median [range] age, 61 [34-90] years; 1006 [99.6%] female and 4 [0.4%] male), 138 (13.7%) had a high nodal burden (Table). In 846 patients with 1 SLN macrometastasis, 83 (9.8%) had ≥pN2, while in 164 with 2 SLN macrometastases, 55 had ≥pN2 (33.5%) (*P* < .001). The median (range) tumor size was significantly smaller in patients with a low nodal burden (19.0 mm [1.1-136.0] vs 25.0 mm [4.0-155.0]; *P* < .001). Additional SLN micrometastases were less common in the low nodal burden group (84 of 872 [9.6%] vs 22 of 138 [15.9%]; *P* = .07), and SLN extracapsular extension significantly less prevalent (296 of 868 [34.1%] vs 66 of 137 [48.2%]; *P* = .002). No difference was seen between low vs high nodal burden for lymphovascular invasion in the primary tumor (242 of 870 [27.8%] vs 38 of 137 [27.7%]; *P* > .99).

**Table.** Baseline Characteristics of Patients

Characteristic	No. (%)			*P* value	Trend test
	Low nodal burden, pN1 (n = 872)	High nodal burden, ≥pN2 (n = 138)	Overall (N = 1010)		
Sex					
Female	868 (99.5)	138 (100)	1006 (99.6)	.95	NA
Male	4 (0.5)	0	4 (0.4)		
Age at randomization, y					
<40	19 (2.2)	2 (1.4)	21 (2.1)	.92	>.99
40-49	137 (15.7)	24 (17.4)	161 (15.9)		
50-64	346 (39.7)	55 (39.9)	401 (39.7)		
65-74	262 (30.0)	38 (27.5)	300 (29.7)		
≥75	108 (12.4)	19 (13.8)	127 (12.6)		
Mean (SD)	61.19 (11.49)	60.91 (11.43)	61.15 (11.47)	.79	NA
Median (range)	61.00 (34.00-90.00)	60.00 (34.00-88.00)	61.00 (34.00-90.00)	NA	NA
T stage					
pT1	501 (57.5)	56 (40.6)	557 (55.1)	<.001	<.001
pT2	331 (38.0)	61 (44.2)	392 (38.8)		
pT3	40 (4.6)	21 (15.2)	61 (6.0)		
Tumor size, mm					
Mean (SD)	22.78 (15.61)	31.14 (21.67)	23.92 (16.81)	<.001	NA
Median (range)	19.00 (1.10-136.00)	25.00 (4.00-155.00)	19.00 (1.10-155.00)	NA	NA
Tumor type					
NST	673 (77.2)	92 (66.7)	765 (75.7)	.01	NA
ILC	170 (19.5)	42 (30.4)	212 (21.0)		
Other/mixed	29 (3.3)	4 (2.9)	33 (3.3)		
Histological grade (NHG)					
1	175 (20.2)	31 (22.5)	206 (20.5)	.78	.48
2	559 (64.5)	88 (63.8)	647 (64.4)		
3	133 (15.3)	19 (13.8)	152 (15.1)		
Missing	5 (0.6)	0	5 (0.5)		
Lymphovascular invasion					
No	628 (72.2)	99 (72.3)	727 (72.2)	>.99	NA
Yes	242 (27.8)	38 (27.7)	280 (27.8)		
Missing	2 (0.2)	1 (0.7)	3 (0.3)		
Breast surgery performed					
Breast-conserving surgery	592 (67.9)	73 (52.9)	665 (65.8)	<.001	NA
Mastectomy	280 (32.1)	65 (47.1)	345 (34.2)		
Suspicious lymph nodes on ultrasonography					
No	760 (87.2)	113 (81.9)	873 (86.4)	.12	NA
Yes	112 (12.8)	25 (18.1)	137 (13.6)		
SLNs removed, No.					
1-2	618 (70.9)	102 (73.9)	720 (71.3)	.57	.35
3-4	221 (25.3)	33 (23.9)	254 (25.1)		
>4	33 (3.8)	3 (2.2)	36 (3.6)		
Mean (SD)	2.10 (1.15)	2.03 (1.03)	2.09 (1.13)	.47	NA
Median (range)	2.00 (1.00-9.00)	2.00 (1.00-6.00)	2.00 (1.00-9.00)	NA	NA
SLN macrometastases, No.					
1	763 (87.5)	83 (60.1)	846 (83.8)	<.001	NA
2	109 (12.5)	55 (39.9)	164 (16.2)		
SLN micrometastases, No.					
0	788 (90.4)	116 (84.1)	904 (89.5)	.07	.02
1	79 (9.1)	20 (14.5)	99 (9.8)		
2	5 (0.6)	2 (1.4)	7 (0.7)		
Total SLN metastases, No.					
1	685 (78.6)	67 (48.6)	752 (74.5)	<.001	<.001
2	176 (20.2)	63 (45.7)	239 (23.7)		
3	11 (1.3)	8 (5.8)	19 (1.9)		
Mean (SD)	1.23 (0.45)	1.57 (0.60)	1.27 (0.49)	<.001	NA
Median (range)	1.00 (1.00-3.00)	2.00 (1.00-3.00)	1.00 (1.00-3.00)	NA	NA
SLN ratio (SLN metastases/excised SLN)					
Mean (SD)	0.70 (0.29)	0.85 (0.23)	0.72 (0.29)	<.001	NA
Median (range)	0.67 (0.11-1.00)	1.00 (0.25-1.00)	0.67 (0.11-1.00)	NA	NA
Extracapsular extension SLN					
No	572 (65.9)	71 (51.8)	643 (64.0)	.002	NA
Yes	296 (34.1)	66 (48.2)	362 (36.0)		
Missing	4 (0.5)	1 (0.7)	5 (0.5)		
Total lymph nodes removed, No.					
Mean (SD)	15.09 (6.86)	17.72 (8.00)	15.45 (7.08)	<.001	NA
Median (range)	14.00 (1.00-51.00)	17.00 (4.00-50.00)	14.00 (1.00-51.00)	NA	NA
Metastases, No.					
Mean (SD)	1.52 (0.67)	7.54 (6.00)	2.34 (3.09)	<.001	NA
Median (range)	1.00 (1.00-3.00)	5.00 (4.00-42.00)	1.00 (1.00-42.00)	NA	NA
Final nodal stage					
pN1	872 (100)	0	872 (86.3)	<.001	<.001
pN2	0	108 (78.3)	108 (10.7)		
pN3	0	30 (21.7)	30 (3.0)		

Abbreviations: ILC, invasive lobular carcinoma; NA, not applicable; NHG, Nottingham Histological Grade; NST, invasive breast cancer of no special type; SLN, sentinel lymph node.

#### Invasive Lobular Cancer

ILC constituted 21.0% of the entire luminal *ERBB2*-negative cohort. High nodal burden was more common in ILC than other histopathological tumor types (42 of 212 [19.8%] vs 96 of 798 [12.0%]; *P* = .005) (eTable in Supplement 1). The median (range) tumor size was significantly larger in patients with ILC (32.0 mm [6.0-155.0] vs 18.0 mm [1.1-97.0]; *P* < .001).

### Prediction Models

#### Luminal *ERBB2*-Negative Cohort

**Figure 1.**
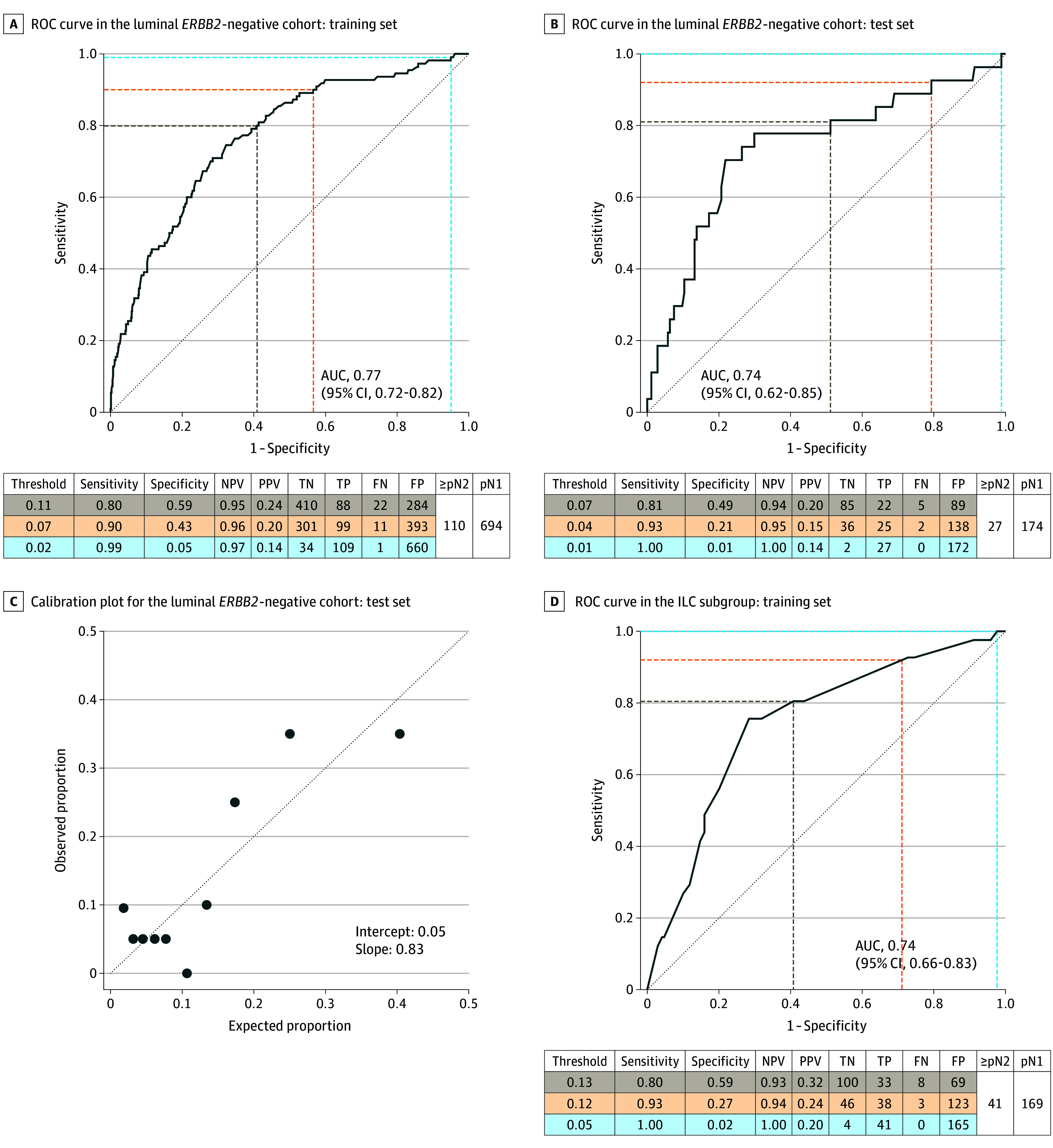
Discrimination and Calibration of the Prediction Models for High Nodal Burden Threshold refers to the predicted probability of high nodal burden (≥pN2). In the calibration plot, the dashed oblique reference line indicates perfect calibration with an intercept of 0 and a slope of 1. The cluster of points in the lower left corner of the figure indicates that observed, as well as predicted, risk of high nodal burden is low for most patients in the cohort. In addition, the absence of a linear trend at this end of the risk spectrum suggests that the discrimination is poor for low-risk patients. However, the points in the upper right corner indicate that the model might be useful for identification of patients at high risk of high nodal burden, which was the aim of the present work. AUC indicates area under the ROC curve; FN, false negative; FP, false positive; N, nodal status; ILC, invasive lobular carcinoma; NPV, negative predicted value; PPV, positive predicted value; TN, true negative; TP, true positive; ROC, receiver operating characteristic curve.

**Figure 2.**
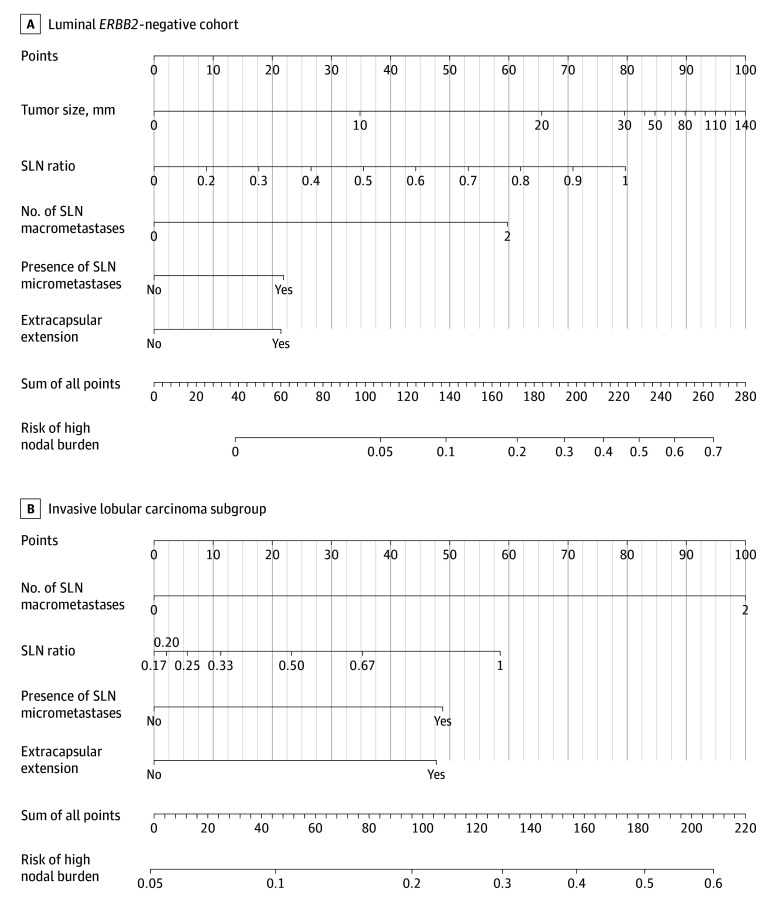
Nomogram for Predicting the Probability of High Nodal Burden SLN ratio refers to (SLN micrometastases + SLN marcrometastases) / (all removed lymph nodes at SLN biopsy). SLN indicates sentinel lymph node.

Two vs 1 SLN macrometastases, higher SLN ratio, additional SLN micrometastases, SLN extracapsular extension, and larger tumor size were independently associated with high nodal burden and selected for the multivariable prediction model (eAppendix 2 in Supplement 1), which reached an AUC of 0.77 (95% CI, 0.72-0.82) in the training set and 0.74 (95% CI, 0.62-0.85) in the test set (Figure 1A and B). The calibration plot showed that the mean predicted probabilities of high nodal burden did not deviate systematically from observed fractions in deciles based on the predicted probabilities of high nodal burden in the test set (minor deviations from the dashed identity line, which indicates perfect calibration). This was also supported by the summary measures calibration intercept 0.05 and calibration slope 0.83, which are close to the optimal values 0 and 1.00, respectively, for a perfectly calibrated model (Figure 1C). At a sensitivity threshold of 80% or greater, 111 of the 201 patients in the test set (55.2%) were identified as high risk (Figure 1B). While this proportion seems high compared to the observed prevalence for ≥pN2 of 13.4%, the negative predictive value was 94.4% (85/90), correctly classifying all but 5 patients as low risk at this threshold. The model is visualized in a nomogram (Figure 2A).

#### Lobular Subgroup

In the ILC subgroup, 2 SLN macrometastases vs 1 had an odds ratio (OR) of 4.96 (95% CI, 2.17-11.47) in the final multivariable model for high vs low nodal burden (eAppendix 2 in Supplement 1). All independent factors identified in the luminal *ERBB2*-negative cohort, apart from tumor size, were associated with high nodal burden in ILC also and were included in the lobular prediction model with an AUC of 0.74 (95% CI, 0.66-0.83) in the development set (Figure 1D). The model is visualized in a nomogram (Figure 2B).

### RFS

**Figure 3.**
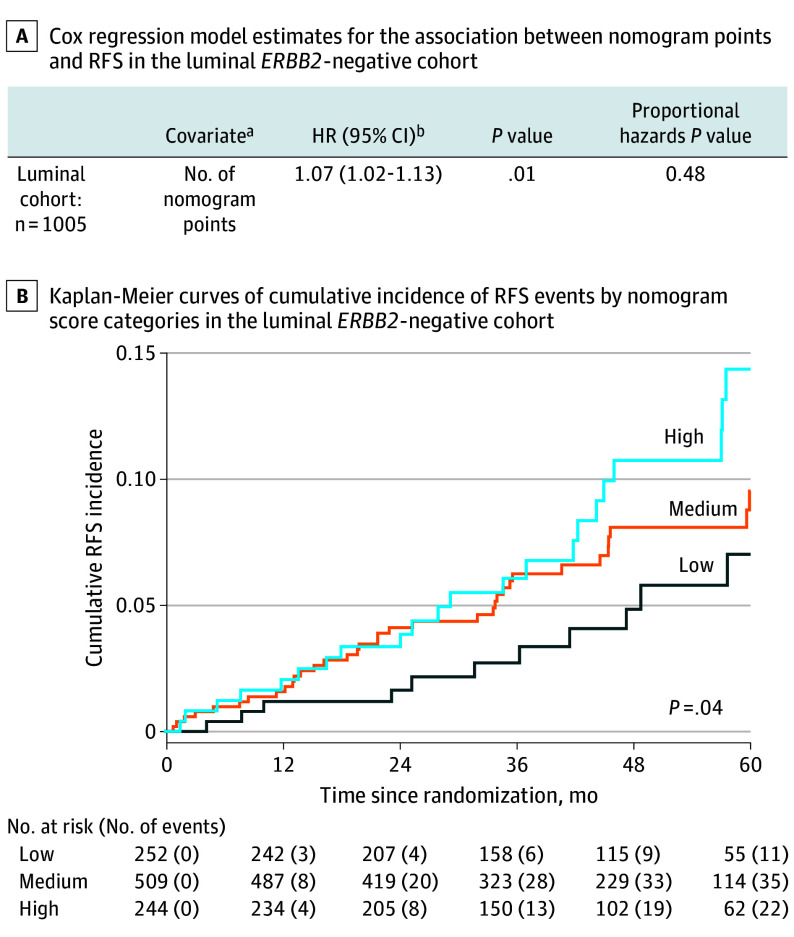
Prognostic Value of the High Nodal Burden Prediction Model Patients were followed up from randomization to the date of first recurrence or death, defined according to the Standardized Definitions for Efficacy End Points (STEEP) criteria or, for event-free patients, to the date of last follow-up. Contralateral breast cancer was not considered a recurrence-free survival (RFS) event. Follow-up times for recurrence-free and alive participants were censored at the date of last visit. No follow-up times were censored for other reasons. The proportional hazards assumption was fulfilled for both Cox models (*P* = .48 in model with continuous score and *P* = .41 with score categories). Risk groups were created by dividing the total nomogram score into 3 groups: low (quartile 1), medium (quartiles 2-3), and high (quartile 4). The hazard ratio (HR) was calculated for a 10-unit nomogram score increase. ^a^Different spline functions were assessed using Akaike Information Criterion (AIC), but a linear model for total points gave the best fit to the data after adjustment for model complexity. ^b^Interpretation of this HR: for each 10-step increase of total nomogram points, the incidence of RFS events increases with 7%.

To investigate the prognostic potential of the prediction model, the total nomogram score was related to RFS in the luminal *ERBB2*-negative cohort. Cox regression analysis revealed that a 10-unit increase in the score corresponded to a 7% increase in the incidence of RFS events (hazard ratio [HR], 1.07; 95% CI, 1.02-1.13; *P* = .01) (Figure 3A). When categorizing the score into quartiles, the HR for the high-risk group (quartile 4) vs the low-risk group (quartile 1) was 2.11 (95% CI, 1.02-4.35; *P* = .04) and the HR for the medium-risk group (quartiles 2-3) vs quartile 1 was 1.59 (95% CI, 0.81-3.14; *P* = .18). The corresponding Kaplan-Meier estimates of cumulative incidence of RFS events are shown in Figure 3B.

## Discussion

In this diagnostic/prognostic study, the predictive models and corresponding nomograms for high nodal burden (≥pN2) in patients with 1 or 2 SLN macrometastases and luminal *ERBB2*-negative breast cancer and ILC, respectively, may help select patients for intensified adjuvant therapies while avoiding CALND. At a threshold corresponding to a sensitivity of at least 80%, all but 5 low-risk patients were correctly classified, corresponding to a negative predictive value of 94%. In the luminal *ERBB2*-negative cohort, the predicted probability of high nodal burden (≥pN2) was also prognostic of RFS, underscoring the clinical relevance of the prediction model as an alternative to CALND.

A minority of patients with 1 or 2 macrometastases on SLN biopsy have a high nodal burden: in the American College of Surgeons Oncology Group Z0011 (ACOSOG Z0011) trial,^1^ 47 of 343 (13.7%) had 4 or more metastatic ALNs; in the European Organisation for Research and Treatment of Cancer 10981-22023 (AMAROS) trial,^3^ 52 of 672 (8%) had 4 or more positive additional nodes (besides SLN metastasis); and in the Optimal Treatment of the Axilla—Surgery or Radiotherapy After Positive Sentinel Lymph Node Biopsy in Early-Stage Breast Cancer (OTOASOR) trial,^34^ 54 of 244 (22%) had ≥pN2a. Despite the presence of ≥pN2 disease in these trials, the omission of CALND did not increase long-term locoregional recurrence rates. However, there are currently no data from randomized clinical trials comparing CALND vs its omission in a population of patients with verified ≥pN2 disease. The proportion of ≥pN2 status ranges from 3.5% to 16% in cN0 breast cancer.^35,36^ Thus, the therapeutic effect of CALND is debatable. Here we present a tool for procuring similar diagnostic information considering multiple risk factors of ≥pN2.

### Prediction Models—Comparison to Previous Studies

In the multicenter tool for risk prediction of 4 or more ALN metastases in SLN-positive breast cancer developed by Meretoja et al,^28^ based on retrospective data from 675 patients, the AUC reached 0.77 (95% CI, 0.74-0.81) in the external validation using a retrospective cohort (n = 760). The included variables were site prevalence of ≥pN2, number of metastatic and benign SLNs, tumor size, and extracapsular extension. Murata et al^25^ presented a model for prediction of high nodal burden based on 804 patients who were cN0 SLN positive. The AUC was 0.89 (95% CI, 0.84-0.93) in the test set, with good discrimination; however, as this was a retrospective single-institution study, external validation is needed. In another prediction model by Kim et al,^26^ lymphovascular invasion, extranodal extension, T stage, and a high SLN ratio were independently associated with high nodal burden in a retrospective analysis of 1437 patients. The AUC was 0.82 in the test set (no CI provided; no external validation). In a nomogram by Yang et al^24^ incorporating the number of SLN metastases, extracapsular extension, pT stage, and tumor location in the breast in 1480 patients, the AUC was 0.80 (95% CI, 0.76-0.85) in the external validation set. In a small study by Corsi et al^27^ (n = 271), SLN ratio, extranodal extension, and multifocal disease were predictors of more than 2 metastatic ALNs, with an AUC of 0.74 (no CI provided; no external validation).

The prevalence of high nodal burden in the above-mentioned studies ranged from 5.7% to 20.7% (compared to 13.7% in this study). While the models based on retrospective cohorts by Meretoja et al and Yang et al were externally validated, none have been evaluated in terms of recurrence or survival analyses.

### ILC

ILC is a distinct biological entity which warrants specific focus. The presented prediction model for ILC is, to our knowledge, the first model for high nodal burden in ILC and 1 or 2 SLN macrometastases. Studies are rarely analyzed by histopathological tumor type,^37^ and treatment recommendations usually address NST and ILC as 1 entity.^4^ Only in recent years, ILC-specific clinical trials have been initiated.^16^

Tumor size was selected in the luminal *ERBB2*-negative cohort but not in the ILC subgroup. Lee et al^36^ and Rivers et al^38^ found tumors greater than 20 mm to be associated with high nodal burden. However, in our study larger tumor size was independently associated with high nodal burden in the luminal *ERBB2*-negative but not the ILC subgroup. Considering the suggested threshold at greater than 20 mm, the significantly larger tumor size in ILC compared to other histopathological tumor types (median, 32 mm vs 18 mm) might explain why tumor size was not a strong independent predictive factor in the ILC subgroup.

Imaging is highly indicative of high nodal burden and can rule out stage ≥pN2 breast cancer with a negative predictive value of 96%.^11,39^ The sensitivity of axillary ultrasonography for predicting ≥3 metastatic ALN is, however, lower in ILC than in NST.^20^ Despite ILC being associated with more ALN metastases,^40,41^ nodal burden is more often underestimated preoperatively.^42^ Other imaging modalities, such as magnetic resonance imaging or ^18^F-fluorodeoxyglucose positron emission tomography/computed tomography, are of limited clinical value.^42,43,44^ These limitations underline the need for prediction models developed exclusively in ILC.

Altogether, ILC poses diagnostic staging challenges, and the risk of occult ALN involvement remains challenging. The use of a decision support tool might thus be more important in ILC than in NST.

### Strengths and Limitations

A major strength of this study is that it is based on meticulously curated prospectively collected data from a large randomized clinical trial. Moreover, we address both global performance measures of discrimination and calibration. In addition, applied sensitivity cutoffs are derived from clinical reasoning rather than being data driven. We exclusively incorporated routinely accessible variables, available at the intended time point for using the nomogram. To further enable clinical use, a nomogram is presented for each target population. While prediction models were not developed to predict RFS, we show clinical validity by differentiation between prognostic strata based on nomogram scores. Longer follow-up will be necessary to monitor separation of RFS curves given the potential of luminal breast cancer to recur late.

The foremost limitation of this study is the lack of validation in an external cohort and the fact that no internal validation was conducted for the ILC subgroup due to limited sample size. Although the inclusion criteria in SENOMAC are considered broad, generalization beyond this population should be used with caution. Another limitation is the absence of some variables of putative relevance for modeling, such as mode of detection (eg, screening or clinically detected), race, ethnicity, and body mass index. Progesterone receptor status and proliferation rate (Ki67) were not considered because of poor standardization and varying availability across centers. The 21-gene Oncotype DX Breast Recurrence Score may inform decisions on adjuvant chemotherapy in postmenopausal luminal *ERBB2*-negative breast cancer with 1 to 3 metastatic ALNs without CALND. However, the clinical value of Oncotype DX for guidance of adjuvant abemaciclib is not yet determined, and a nomogram can be applied irrespective of the availability of genomic tests.^45^ In the SENOMAC trial, multigene signatures were not registered. While acknowledging this as a limitation, we emphasize that the presented models are clinically feasible also in economically diverse conditions and not dependent on the availability of tissue for analysis. No imaging-derived variables were considered for the models, enabling application in routine clinical settings where axillary imaging is not part of diagnostic workup for patients with cN0 T1-T2 breast cancer eligible for upfront surgery.^11,46^

## Conclusions

The presented model predicts high nodal burden in patients with luminal *ERBB2*-negative T1-T3 breast cancer and 1 or 2 SLN macrometastases with good performance measures. Use of the nomogram may facilitate systemic treatment decisions without exposing patients to CALND, thus minimizing the risk of arm morbidity. External validation is necessary.

## Notes

Supplement 1. eFigure. Patient Flow Chart eAppendix 1. Prediction Model – Variable Selection eAppendix 2. Multivariable Logistic Regression Model eTable. Baseline Characteristics of the Included Patients Stratified on Invasive Lobular Carcinoma (ILC) vs Other Histopathological Tumor Types

Supplement 2. Data sharing statement
