# Supplementary material for: Prediction of High Nodal Burden in Patients With Sentinel Node–Positive Luminal ERBB2-Negative Breast Cancer
Source: JAMA Surg. 2024 Sep 25;159(12):1393–403. doi: 10.1001/jamasurg.2024.3944 (PMC11425194; doi:10.1001/jamasurg.2024.3944)
Supplement: Supplement 1. — eFigure. Patient Flow Chart eAppendix 1. Prediction Model – Variable Selection eAppendix 2. Multivariable Logistic Regression Model eTable. Baseline Characteristics of the Included Patients Stratified on Invasive Lobular Carcinoma (ILC) vs Other Histopathological Tumor Types [file jamasurg-e243944-s001.pdf]

Supplemental Online Content

Skarping I, Pär-Ola Bendahl PO, Szulkin R, et al. Prediction of high nodal burden in patients with sentinel node–positive luminal erbb2-negative breast cancer. *JAMA Surg*. Published online September 25, 2024. doi:10.1001/jamasurg.2024.3944

eFigure. Patient Flow Chart

eAppendix 1. Prediction Model – Variable Selection

eAppendix 2. Multivariable Logistic Regression Model

eTable. Baseline Characteristics of the Included Patients Stratified on Invasive Lobular Carcinoma (ILC) vs Other Histopathological Tumor Types

This supplemental material has been provided by the authors to give readers additional information about their work.

© 2024 Skarping I et al. *JAMA Surgery.*

eFigure. Patient Flow Chart

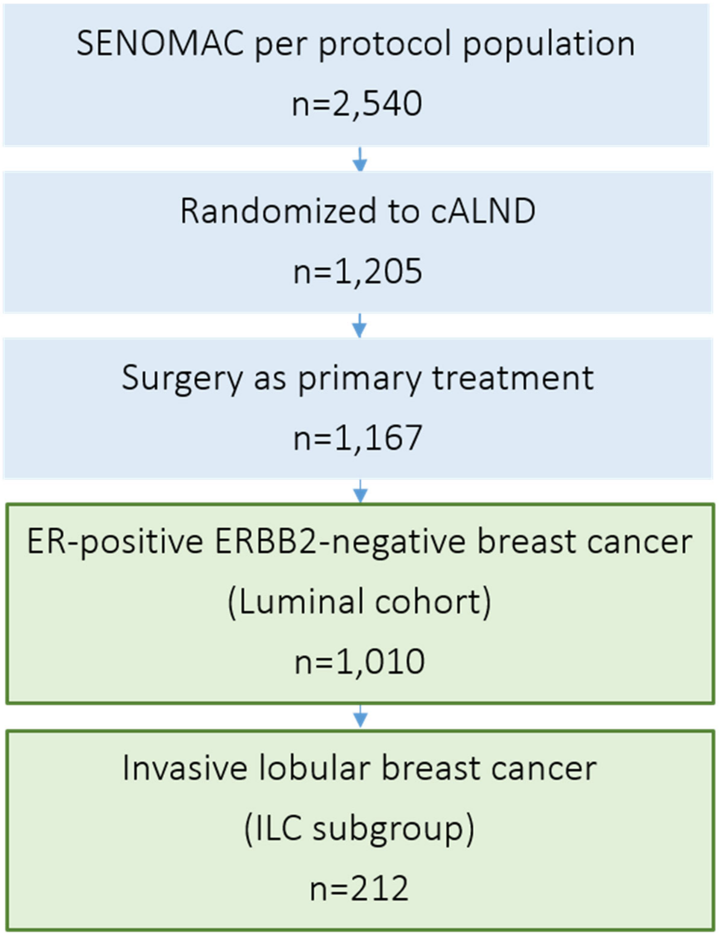

© 2024 Skarping I et al. *JAMA Surgery*.

eAppendix 1. Prediction Model – Variable Selection

a) A priori Identified Candidate Predictors

	**Luminal ERBB2-negative cohort**		**Lobular breast cancer subgroup**	
	Included	Reason for exclusion	Included	Reason for exclusion
Age, years [continuous]		Selected in only 229 of 1000 bootstrap samples		Selected in only 406 of 1000 bootstrap samples
Tumor size, mm [continuous]	Selected in 973 of 1000 bootstrap samples*			Selected in only 223 of 1000 bootstrap samples
Number of SLN macrometastases [1 or 2]	Selected in 1000 of 1000 bootstrap samples		Selected in 985 of 1000 bootstrap samples	
Presence of micrometastases [Yes/No]	Selected in 502 of 1000 bootstrap samples		Selected in 549 of 1000 bootstrap samples	
Suspicious lymph nodes on ultrasound [Yes/No]		Selected in only 161 of 1000 bootstrap samples		Selected in only 272 of 1000 bootstrap samples
Size of SLN metastases [continuous]		Large proportion of missing values first years of inclusion. In a clinical context, absolute number of micro- and macrometastases alongside ratio metastatic SLNs/extracted SLNs is used, not the size per se.		Missing values first years of inclusion
Extracapsular extension of metastatic SLNs [Yes/No]	Selected in 800 of 1000 bootstrap samples		Selected in 734 of 1000 bootstrap samples	
Vascular invasion (in tumor) [Yes/No]		Selected in only 131 of 1000 bootstrap samples		Selected in only 179 of 1000 bootstrap samples
Histopathological type [ILC vs other/mixed histopathological subtypes]		Selected in only 179 of 1000 bootstrap samples		ILC inclusion criteria
Multifocality [Yes/No]		Selected in only 191 of 1000 bootstrap samples		Selected in only 314 of 1000 bootstrap samples
Nottingham histological grade [3 vs 1 or 2]		Selected in only 174 of 1000 bootstrap samples		Not relevant for ILC (most ILCs are grade 2)

© 2024 Skarping I et al. *JAMA Surgery.*

Nottingham histological grade [2 or 3 vs 1]		Selected in only 366 of 1000 bootstrap samples		Not relevant for ILC (most ILCs are grade 2)
SLN ratio (SLN micro- and macrometastases/extracted SLNs) [continuous]	Selected in 1000 of 1000 bootstrap samples		Selected in 523 of 1000 bootstrap samples	

*A bootstrap sample is a random sample of the same size as original cohort drawn with replacement. Hence, some samples will not be represented in a specific bootstrap sample whereas other samples will be represented by 1, 2, 3, or even more copies. (ref: Efron, B, (1979). "Bootstrap methods: Another look at the jackknife" *The Annals of Statistics* 7(1): 1-26 doi: 10.1214/aos/1176344552)

Abbreviations: ERBB2: Erb-B2 receptor tyrosine kinase 2; SLN: sentinel lymph node; ILC: invasive lobular carcinoma

© 2024 Skarping I et al. *JAMA Surgery.*

b) Prediction Model Development

For the development of the luminal ERBB2-negative prediction model, 80% of the luminal cohort was utilized (training set). The remaining 20% was set aside for internal validation (test set). The random 80/20 split was stratified by outcome to guarantee the same prevalence of outcome in the training and the test set. The ILC subgroup was considered too small to be split. Hence, all the ILC patients were used to develop the prediction model for this cohort, a model which was therefore not internally validated.

A set of candidate predictors was compiled based on the literature and expert knowledge. Binary coding (0/1) was used for categorical and/or categorized predictors with two levels. The ordinal variable Nottingham histological grade with levels 1, 2, and 3 was transformed to two dummy variables corresponding to the two possible dichotomizations of the variable, i.e. 1 vs 2/3 and 1/2 vs 3. This dummy coding would be interpretable if zero, one, or two of the dummy variables were included in the final model. Restricted cubic splines were used to evaluate the support for nonlinear effects of the continuous variables on the binary outcome in the development cohort. In addition to the linear coding (no splines) the number of knots was varied from 3 to 6 and the corresponding Akaike Information criterion (AIC) values from unadjusted logistic regression models were calculated. An AIC drop of more than 2 units was considered large enough to motivate increased model complexity. However, for one variable (ratio of metastatic and excised SLNs) in the training set of the luminal ERBB2-negative cohort, the model with increased complexity was discarded despite lower AIC. A linear relationship between this variable and the outcome was considered clinically more plausible.

For each of the two cohorts (the luminal development cohort and the ILC subgroup) prediction models were developed for each of 1000 bootstrap samples using multivariable logistic regression with backward selection. The p-value cut-off for exclusion was set to 0.157, corresponding to minimization of AIC. Final subsets of predictors were identified based on the number of times each predictor was included over the 1000 bootstrap samples. A cut-off of 500, i.e. selection in at least 50% of the bootstrap samples, was used. The final models were fitted using only these variables.

© 2024 Skarping I et al. *JAMA Surgery.*

eAppendix 2. Multivariable Logistic Regression Models

a) Prediction Model for the Whole Luminal ERBB2-Negative Cohort. Coefficients Estimated in the Training Set (n=804)

Equation:

Variable	β	SE
Intercept	-5.73524002 (β_0)	0.68027848
Number of SLN macrometastases, X_1	1.43712354 (β_1)	0.23771007
Prescence of SLN micrometastases [1=Yes, 0=No], X_2	0.52566713 (β_2)	0.31463225
SLN ratio [continuous]*, X_3	2.12914128 (β_3)	0.48082934
Tumor size [continuous], X_4	0.08373917 (β_4)	0.02969195
Spline transformed tumor size [continuous], X_5 (defined below)	-0.08849662 (β_5)	0.03847920
Extracapsular extension [1=Yes, 0=No], X_6	0.51443264 (β_6)	0.22369397

Abbreviations: SLN, sentinel lymph node

The probability *p* of heavy nodal burden according to this model is:

$p = \exp(LP)/(1+\exp(LP))$

where LP is the linear predictor

$LP = \beta_0 + \beta_1 * X_1 + \beta_2 * X_2 + \beta_3 * X_3 + \beta_4 * X_4 + \beta_5 * X_5 + \beta_6 * X_6$

The definition of the spline transformed tumor size X_5 (second basis function of the natural cubic spline) is as follows:

Let $k_1 = 10$, $k_2 = 19$, and $k_3 = 40$ mm be the positions of the three knots of the restricted cubic spline for tumor size.

Next, define the following three cubic transformation of tumor size:

$a = ((X_4-k_1)*((X_4-k_1)>0))^3$

$b = ((X_4-k_2)*((X_4-k_2)>0))^3$

$c = ((X_4-k_3)*((X_4-k_3)>0))^3$

Note that $(X_4-k_i)>0$ is a dummy(1/0). Next combine the three cubic transformations:

$d = a-b*(k_3-k_1)/(k_3-k_2)+c*(k_2-k_1)/(k_3-k_2)$

And finally normalize to get:

$X_5 = d/((k_3-k_1)^2)$

© 2024 Skarping I et al. *JAMA Surgery.*

Variables included in the model for the whole luminal ERBB2-negative cohort.

Variable		n (events[a])	Unadjusted OR (95% CI)	*P* value	n (events[a])	Adjusted OR (95% CI)	*P* value
Number of SLN macrometastases	1	673 (66)	1.00 (reference)	<0.001	669 (65)	1.00 (reference)	<0.001
	2	135 (45)	4.60 (2.96-7.12)		135 (45)	4.21 (2.64- 6.71)	
Prescence of SLN micrometastases	No	724 (94)	1.00 (reference)	0.070	721 (93)	1.00 (reference)	0.095
	Yes	84 (17)	1.70 (0.93-2.96)		83 (17)	1.69 (0.89- 3.08)	
SLN ratio [continuous]*			10.52 (4.60-25.68)	<0.001		8.41 (3.38-22.41)	<0.001
Tumor size [continuous]			**	<0.001[b]		***	<0.001[b]
Extracapsular extension	No	521 (59)	1.00 (reference)	0.009	521 (59)	1.00 (reference)	0.021
	Yes	283 (51)	1.72 (1.14-2.58)		283 (51)	1.67 (1.08- 2.59)	

Abbreviations: CI, confidence interval; OR, odds ratio; SLN, sentinel lymph node

[a]High nodal burden[b]*P* value from likelihood ratio test with spline function removed from the mode

*SLN ratio defined as: $\frac{SLN\ \text{micrometastases+SLN marcrometastases}}{all\ removed\ lymphnodes\ at\ SLN\ biopsy}$

**Spline function of tumor size in univariable (unadjusted) model

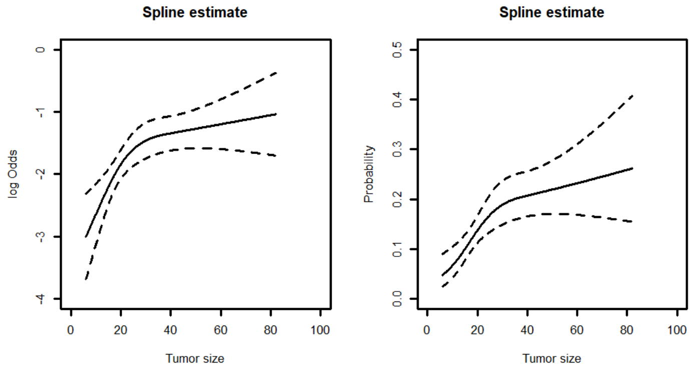

***Spline function of tumor size vs log odds (left panel) and probability (right panel) of ≥pN2, multivariable model

© 2024 Skarping I et al. *JAMA Surgery.*

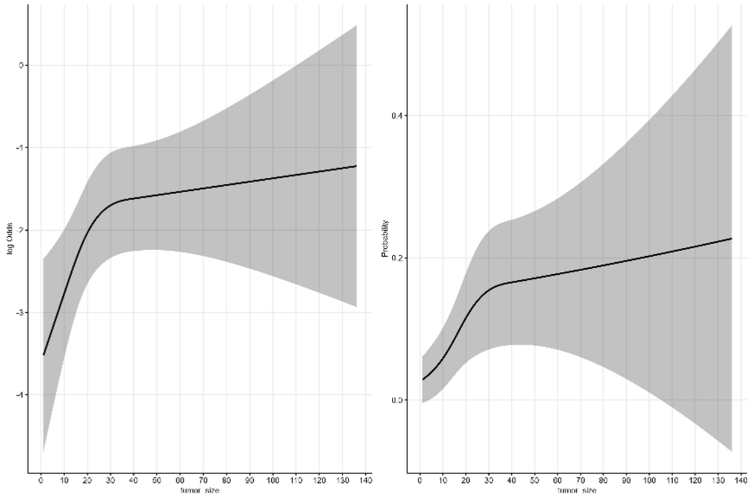

© 2024 Skarping I et al. *JAMA Surgery*.

b) Prediction Model for the Invasive Lobular Carcinoma (ILC) Subgroup (n=210)

Equation:

**Variable**	**β**	**SE**
Intercept	-3.1133535	0.6881553
Number of SLN macrometastases	1.6004329	0.4225479
Prescence of SLN micrometastases [1=Yes, 0=No]	0.7799631	0.5448775
SLN ratio [continuous]*	1.1283853	0.7716655
Extracapsular extension [1=Yes, 0=No]	0.7632402	0.3788890

Abbreviations: SLN, sentinel lymph node

Variables included in the model for the ILC subgroup

**Variable**		**n (events[a])**	**Unadjusted OR (95% CI)**	***P* value**	**n (events[a])**	**Adjusted OR (95% CI)**	***P* value**
Number of SLN macrometastases	1	173 (25)	1.00 (reference)	<0.001	171 (24)	1.00 (reference)	<0.001
	2	39 (17)	4.57 (2.13-9.84)		39 (17)	4.96 (2.17-11.47)	
Prescence of SLN micrometastases	No	188 (36)	1.00 (reference)	0.500	186 (35)	1.00 (reference)	0.152
	Yes	24 (6)	1.41 (0.48-3.63)		24 (6)	2.18 (0.70-6.14)	
SLN ratio [continuous]*			4.79 (1.29-20.28)	0.025		3.09 (0.71-15.04)	0.144
Extracapsular extension	No	141 (22)	1.00 (reference)	0.043	141 (22)	1.00 (reference)	0.044
	Yes	69 (19)	2.06 (1.02-4.13)		69 (19)	2.15 (1.02-4.53)	

Abbreviations: CI, confidence interval; OR, odds ratio; SLN, sentinel lymph node

[a]High nodal burden

*SLN ratio defined as: $\frac{SLN\ \text{micrometastases+SLN marcrometastases}}{all\ removed\ lymphnodes\ at\ SLN\ biopsy}$

© 2024 Skarping I et al. *JAMA Surgery.*

eTable. Baseline Characteristics of the Included Patients Stratified on Invasive Lobular Carcinoma (ILC) vs Other Histopathological Tumor Types

	ILC (n=212)	Other/Mixed Histopathological Tumor Types (n=798)	Overall (n=1,010)	*P* value	Trend test
**Sex**					
Female	212 (100%)	794 (99.5%)	1006 (99.6%)	0.676	
Male	0 (0%)	4 (0.5%)	4 (0.4%)		
**Age at randomization**					
<40	2 (0.9%)	19 (2.4%)	21 (2.1%)	0.080	0.009
40-49	29 (13.7%)	132 (16.5%)	161 (15.9%)		
50-64	74 (34.9%)	327 (41.0%)	401 (39.7%)		
65-74	75 (35.4%)	225 (28.2%)	300 (29.7%)		
75+	32 (15.1%)	95 (11.9%)	127 (12.6%)		
Mean (SD)	63.00 (11.51)	60.66 (11.42)	61.15 (11.47)	0.008	
Median [Min-Max]	65.00 [35.00-88.00]	61.00 [34.00-90.00]	61.00 [34.00-90.00]		
**T-stage**					
pT1	67 (31.6%)	490 (61.4%)	557 (55.1%)	<0.001	<0.001
pT2	100 (47.2%)	292 (36.6%)	392 (38.8%)		
pT3	45 (21.2%)	16 (2.0%)	61 (6.0%)		
**Tumor size, mm**					
Mean (SD)	37.15 (25.89)	20.40 (11.01)	23.92 (16.81)	<0.001	
Median [Min-Max]	32.00 [6.00-155.00]	18.00 [1.10-97.00]	19.00 [1.10-155.00]		
**Tumor type**					
NST	0 (0%)	765 (95.9%)	765 (75.7%)	<0.001	
Other/Mixed	0 (0%)	33 (4.1%)	33 (3.3%)		
ILC	212 (100%)	0 (0%)	212 (21.0%)		
**Histological grade (NHG)**					
Grade 1	22 (10.4%)	184 (23.2%)	206 (20.5%)	<0.001	0.568
Grade 2	175 (82.5%)	472 (59.5%)	647 (64.4%)		
Grade 3	15 (7.1%)	137 (17.3%)	152 (15.1%)		
Missing	0 (0%)	5 (0.6%)	5 (0.5%)		
**Lymphovascular invasion**					

© 2024 Skarping I et al. *JAMA Surgery.*

	ILC (n=212)	Other/Mixed Histopathological Tumor Types (n=798)	Overall (n=1,010)	*P* value	Trend test
No	185 (87.7%)	542 (68.1%)	727 (72.2%)	<0.001	
Yes	26 (12.3%)	254 (31.9%)	280 (27.8%)		
Missing	1 (0.5%)	2 (0.3%)	3 (0.3%)		
**Breast surgery performed**					
Breast-conserving surgery	95 (44.8%)	570 (71.4%)	665 (65.8%)	<0.001	
Mastectomy	117 (55.2%)	228 (28.6%)	345 (34.2%)		
**Suspicious lymph nodes on ultrasound**					
No	183 (86.3%)	690 (86.5%)	873 (86.4%)	1.000	
Yes	29 (13.7%)	108 (13.5%)	137 (13.6%)		
**Number of SLNs removed**					
1-2	161 (75.9%)	559 (70.1%)	720 (71.3%)	0.231	0.135
3-4	44 (20.8%)	210 (26.3%)	254 (25.1%)		
>4	7 (3.3%)	29 (3.6%)	36 (3.6%)		
Mean (SD)	2.00 (1.07)	2.12 (1.15)	2.09 (1.13)	0.173	
Median [Min-Max]	2.00 [1.00-6.00]	2.00 [1.00-9.00]	2.00 [1.00-9.00]		
**Number of SLN macrometastases**					
1	173 (81.6%)	673 (84.3%)	846 (83.8%)	0.393	
2	39 (18.4%)	125 (15.7%)	164 (16.2%)		
**Number of SLN micrometastases**					
0	188 (88.7%)	716 (89.7%)	904 (89.5%)	0.775	0.769
1	23 (10.8%)	76 (9.5%)	99 (9.8%)		
2	1 (0.5%)	6 (0.8%)	7 (0.7%)		
**Total number of SLN metastases**					
1	149 (70.3%)	603 (75.6%)	752 (74.5%)	0.030	0.353
2	62 (29.2%)	177 (22.2%)	239 (23.7%)		
3	1 (0.5%)	18 (2.3%)	19 (1.9%)		
Mean (SD)	1.30 (0.47)	1.27 (0.49)	1.27 (0.49)	0.353	

© 2024 Skarping I et al. *JAMA Surgery.*

	ILC (n=212)	Other/Mixed Histopathological Tumor Types (n=798)	Overall (n=1,010)	*P* value	Trend test
Median [Min-Max]	1.00 [1.00-3.00]	1.00 [1.00-3.00]	1.00 [1.00-3.00]		
**SLN ratio (SLN metastases/excised SLN)**					
Mean (SD)	0.76 (0.28)	0.71 (0.29)	0.72 (0.29)	0.035	
Median [Min-Max]	1.00 [0.17-1.00]	0.67 [0.11-1.00]	0.67 [0.11-1.00]		
**Extracapsular extension SLN**					
No	141 (67.1%)	502 (63.1%)	643 (64.0%)	0.321	
Yes	69 (32.9%)	293 (36.9%)	362 (36.0%)		
Missing	2 (0.9%)	3 (0.4%)	5 (0.5%)		
**Total number of lymph nodes removed**					
Mean (SD)	15.07 (7.15)	15.55 (7.06)	15.45 (7.08)	0.377	
Median [Min-Max]	14.00 [2.00-50.00]	15.00 [1.00-51.00]	14.00 [1.00-51.00]		
**Number of metastases**					
Mean (SD)	3.17 (5.01)	2.12 (2.28)	2.34 (3.09)	<0.001	
Median [Min-Max]	2.00 [1.00-42.00]	1.00 [1.00-27.00]	1.00 [1.00-42.00]		
**Final nodal stage**					
pN1	170 (80.2%)	702 (88.0%)	872 (86.3%)	<0.001	<0.001
pN2	28 (13.2%)	80 (10.0%)	108 (10.7%)		
pN3	14 (6.6%)	16 (2.0%)	30 (3.0%)		
**Nodal burden**					
Low nodal burden (pN1)	170 (80.2%)	702 (88.0%)	872 (86.3%)	0.005	
High nodal burden (≥pN2)	42 (19.8%)	96 (12.0%)	138 (13.7%)		

Abbreviations: BC, breast cancer; ILC, invasive lobular carcinoma; NST, invasive breast cancer of no special type; SLN, sentinel lymph node

© 2024 Skarping I et al. *JAMA Surgery.*
