# Supplementary material for: Prediction of High Nodal Burden in Patients With Sentinel Node–Positive Luminal ERBB2-Negative Breast Cancer
Source: JAMA Surg. 2024 Sep 25;159(12):1393–403. doi: 10.1001/jamasurg.2024.3944 (PMC11425194; doi:10.1001/jamasurg.2024.3944)
Supplement: Supplement 2. — Data sharing statement [file jamasurg-e243944-s002.pdf]

Data Sharing Statement

Data
Data available: No

Additional Information
Explanation for why data not available: All data are managed by the Clinical Trials Unit at Karolinska University Hospital, Stockholm, Sweden. A dedicated Data Access Committee receives applications by third parts to use data or material collected during this trial and confirms data extraction with the Trial Committee.
